# Synthesis and biological evaluation of thiazole derivatives as *Lb*SOD inhibitors

**DOI:** 10.1080/14756366.2018.1550752

**Published:** 2018-12-27

**Authors:** Camila C. Bitencourt Brito, Hélder Vinicius Carneiro da Silva, Daci José Brondani, Antonio Rodolfo de Faria, Rafael Matos Ximenes, Ivanildo Mangueira da Silva, Julianna F. C. de Albuquerque, Marcelo Santos Castilho

**Affiliations:** a Programa de pós-graduação em Biotecnologia , Universidade Estadual de Feira de Santana, Feira de Santana, BA, Brazil;; b Departamento de Antibióticos, Universidade Federal de Pernambuco, Recife, PE, Brazil;; d Departamento de Farmácia , Universidade Federal de Pernambuco, Recife, PE, Brazil;; c Faculdade de Belo Jardim, Recife, PE, Brazil;; e Faculdade de Farmácia , Universidade Federal da Bahia, Salvador, BA, Brazil

**Keywords:** Leishmania, superoxide dismutase, thiazole derivatives, thermal shift assay

## Abstract

Leishmaniasis is considered as one of the major neglected tropical diseases due to its magnitude and wide geographic distribution. *Leishmania braziliensis*, responsible for cutaneous leishmaniasis, is the most prevalent species in Brazil. Superoxide dismutase (SOD) belongs to the antioxidant pathway of the parasites and human host. Despite the differences between SOD of *Leishmania braziliensis* and human make this enzyme a promising target for drug development efforts. No medicinal chemistry effort has been made to identify *Lb*SOD inhibitors. Herein, we show that thermal shift assays (TSA) and fluorescent protein-labeled assays (FPLA) can be employed as primary and secondary screens to achieve this goal. Moreover, we show that thiazole derivatives bind to *Lb*SOD with micromolar affinity.

## Introduction

Tropical neglected diseases (NTDs) are among the leading causes of mortality in tropical countries around the world^1^ ^,^ ^2^. Leishmaniasis is endemic in 98 countries and South America countries accounting for 90% of worldwide cases of cutaneous leishmaniasis (CL), which is caused mainly by *Leishmania braziliensis* ^3–5^.

The treatment of patients with CL relies on pentavalent antimonial or, as a second choice, pentamidine or amphotericin B, which requires parenteral administration^6^. However, these drugs show either low efficacy or limited safety profile, such as a high incidence of nausea, lethargy, urticaria, hepatotoxicity, and cardiotoxicity^7^ ^,^ ^8^. In addition, resistance to the available drugs is an increasing limitation to the treatment of patients with leishmaniasis^2^ ^,^ ^9^ ^,^ ^10^. Therefore, there is an urgent need to identify novel drugs to fight leishmaniasis.

One of the first steps to achieve this goal is to identify metabolic differences between the parasite and the human host that might be targeted for selective modulation^11^. All aerobic organisms employ reactive oxygen species (ROS) for intercellular signaling and synthesis of important biological substances^12^ ^,^ ^13^. ROS are also a part of the human innate immune response, once they are involved in the recruitment of inflammatory cells to sites of inflammation^14^ and/or infection^12^. For that reason, the protozoan parasites have developed an efficient protection against ROS, whose first step is controlled by superoxide dismutase (SOD, E.C.1.15.1.1), an enzyme responsible for the dismutation of superoxide into hydrogen peroxide and molecular oxygen^5^ ^,^ ^11^. Accordingly, FeSOD activity has proven essential for Leishmania survival in the host^15^.

The superoxide dismutase from *L. braziliensis* presents iron as a metal prosthetic group (FeSOD) whereas the human counterpart employs Cu/Zn (CuZnSOD)^16^ ^,^ ^17^. This difference along their low-sequence similarity suggests that it is possible to inhibit the FeSOD from trypanosomatids selectively^18–21^. ROMERO et al. (2017) have shown that phthalazine derivatives kill promastigotes and amastigotes forms of *Leishmania spp*, by FeSOD inhibition. Besides, MORENO-VIGURI et al. (2016) have reported that arylaminoketone derivatives inhibit FeSOD from trypanosomatids but have little effect over human CuZnSOD.

These results suggest FeSOD from *L. braziliensis* is a promising target for drug design efforts. However, no medicinal chemistry effort to target this protein has been carried out before. Aiming at overcoming this knowledge-gap, this work describes how thermal shift assays (TSA) and fluorescent protein-labeled assays (FPLA) can be employed as primary and secondary HTS-friendly alternatives to indirect-kinetic assays that measure SOD catalytic activity. TSA relies on environment-sensitive fluorophores that reversibly binds to exposed hydrophobic regions when protein unfolds due to temperature increase^22–24^. Whilst FPLA requires covalent bond formation between the protein and the probe^25^. Finally, we show that this screen-counter-screen strategy led to the identification of a micromolar inhibitor of *L. braziliensis* FeSOD.

## Materials and methods

### Chemistry

Chemical reagents and solvents were purchased from Sigma-Aldrich (St. Louis, MO, USA) with analytical grade purity. Melting points were determined in open capillaries on a Buchi apparatus and are uncorrected. Thin-layer chromatography (TLC) was carried out on aluminium-supported silica gel plates (Merck 60 _F254_) with visualization by UV light 254 nm in the appropriated system for each compound. The Infrared spectra (1% KBr, cm^−1^ pellets) were recorded on a Bruker IFS66 spectrophotometer (Billerica, MA, USA), the wave numbers were given in cm^−1^ and are uncorrected. The ^1^H NMR and ^13 ^C NMR spectra were recorded on a VARIAN VNMRS 400-MR (Palo Alto, CA, USA), 400 MHz for ^1^H and 75.4 MHz for ^13 ^C. The ^1^H spectra were recorded in DMSO-*d*_6_ while ^13 ^C spectra were recorded in CDCl_3_ and DMSO using tetramethylsilane (TMS) as the internal standard. The peak and abbreviations were used to indicate multiplicity: s (singlet), d (doublet), dd (double doublet), ddd (double doublet, doublet), t (triplet), and m (multiplet). The chemical shifts were reported in δ units and the coupling constants (J) were reported in Hertz. Mass spectra were recorded on a Varian MAT 711 spectrometer (Palo Alto, CA, USA) 70 eV electron impact.

### Synthesis of 2-aminothiazole derivatives (Ju-436 and Ju-533)

The 2-aminothiazole derivatives were obtained from the mixture of equimolar amounts 0.175 g (2.3 × 10^−3 ^mol) of thiourea dissolved in 15 ml of methanol followed by the addition of 0.513 g (2.3 × 10^−3 ^mol) of substituted acetophenone halide dissolved in 20 ml methanol, to form a white suspension. The reaction was left for 15 min at room temperature under magnetic stirring. After that, the mixture was dissolved in 0.6 ml of HCl (dropwise). The reaction was heated to 90 °C in an oil bath, the pH adjusted between 4 and 5. The reaction time was 2 h. The reaction was monitored by thin layer chromatography. The product was ice-cooled and filtered. The compound was purified by crystallization from methanol.

### 4-(Bromophenyl)thiazol-2-amine (Ju-436)

Chemical formula: C_9_H_7_BrN_2_S, MW 255.1343, Rdt 98%, Rf 0.50 (0.7: 0.3 Hexane/Ethyl acetate) MP 216–217 °C. **Infrared**: 3390–3317 (NH_2_), 3110 (C–H) 1620 (C = N), 1570 (C = C Ar),1185 (C–N), 1058(C–S–C), 815 (Ar) 739 (Ar–Br). **^1^HNMR**: 6.39 (s 2H); 6,99 (s 1H); 7.77–7.74 (d 2H) J = 8.50 Hz; 7.76–7.71 (d 2H) J = 8.50 Hz. **^13^HNMR**: 121.5, 129.5, 135.6, 129.5, 121.5, 123.4, 136.6, 124.8, 115.0, 125.6. **HRMS^+^**, calculated: 252.9561, found: 252.9558.

### 4–(4-Bromophenyl)thiazol-2-amine (Ju-533)

Chemical formula: C_9_H_7_BrN_2_S, molecular weight: 255.1343, yield 92%, Rf = 0.47 (Hexane/Ethyl acetate, 0.6:0.4). MP 202–204 °C. Infrared 3389–3520 (NH_2_), 3116 (C–H) 1614 (C = N), 1575 (C = C Ar),1180 (C–N), 1067 (C–S–C), 817 (Ar) 740 (Ar–Br). **^1^HNMR**: 8.33 (d 2H orto, J = 7.32 Hz); 8.10 (d, 2H meta, J = 7.32 Hz); 8.12 (s 1H CH); 7.76 (d 1H Ar J = 8.1); 7.63 (d 1H Ar J = 8,1); 7.66 (d 1H, J = 8.2). **^13^HNMR**: 148; 124; 126; 140; 178; 161; 130; 132; 129; 137; 163. **HRMS^+^**, calculated: 255.9493, found: 253.9513.

### General procedure for the synthesis of imines (Ju-445-Ju-555)

The 4–(4-bromophenyl)-thiazol-2-imine-arylidene-substituted and 4–(3 or 4-nitrophenyl)-thiazol-2-imine-arylidene-substituted derivatives were synthesized from the mixture of equimolar amounts 2.3 × 1.0^−3 ^mol of 4–(4-bromo, 3- nitro or 4-nitrophenyl)-2-aminothiazole and 2.3 × 1.0 ^−3 ^mol of substituted benzaldehydes. The substituted benzaldehydes were dissolved in 15 ml of MeOH under stirring at room temperature. After that, 0.08 ml of HCl (conc.) dissolved in MeOH was added dropwise. After 5 min. under cold stirring, the starting material (4-bromo, 4-nitro or 3-nitrophenyl)-2-aminothiazole previously dissolved in 15 ml of MeOH was added dropwise. The pH was then adjusted between 4 and 5 and the reaction was warmed to 75 °C and refluxed in an oil bath for 2–24 h. The progress of the reaction was monitored by TLC. At the end, the solvent was reduced to one-half volume and ice-cooled. The precipitate was filtered off and recrystallized from the appropriate solvent.

### *N*-(2,4-dichlorobenzylidene)-4–(4-nitrophenyl)thiazol-2-imine (Ju-445)

Chemical Formula: C_16_H_9_Cl_2_N_3_O_2_S, MW 378.2326, Rdt 55%, Rf = 0.45 (Hex/AcOEt, 0.6:0.4). MP 218.6–220.0 °C. **Infrared**: 3109 (C–H), 1610 (C = N), 1575 (C = C Ar),1189 (C–N), 1055(C–S–C), 810 (Ar), 730 (Ar–Cl). **^1^HNMR**: 8.35 (d 2H orto, J = 7.32 Hz); 8.10 (d 2H meta, J = 7.38 Hz), 8.11 (s 1H CH–S); 8,28 (s 1H CH = N); 8.27 (s 1H Ar); 7.63 (d 1H Ar, J = 8,2); 7.67 (d 1H, J = 8.2) ^13^ **CNMR**: 124, 130, 135, 129, 121, 124, 111, 152, 173, 179, 133, 131, 137, 136, 130, 129. **HRMS^+^**, calculated: 376.9793, found: 378.9633.

### *N*-(4-bromobenzylidene)-4–(4-nitrophenyl)thiazol-2-imine (Ju-450)

Chemical Formula: C_16_H_10_BrN_3_O_2_S, MW 388.2385, Rdt 45%, Rf = 0.54 (Hex/AcOEt, 0.6:0.4): MP 262 °C. **Infrared** 3112 (C–H), 1613 (C = N), 1575 (C = C Ar), 1179 (C–N), 1061(C–S–C), 815 (Ar), 737 (Ar–Br). **^1^HNMR:** 8.33 (d 2H orto, J = 7.32 Hz); 8.10 (d, 2H meta, J = 7.32 Hz), 8.11 (s 1H CH); 7.74 (d 1H Ar J = 8.1) 7.63 (d 1H Ar J = 8,2); 7.67 (d 1H, J = 8.2) ^13^ **CNMR:** 148; 126; 127; 140; 175; 3. 161; 129; 133; 129; 137; 162. **HRMS^+^**, calculated: 386.9677, found: 388.9557.

### *N*-(3,4-dibromobenzylidene)-4–(4-nitrophenyl)thiazol-2-imine (Ju-480)

Chemical Formula: C_16_H_9_Br_2_N_3_O_2_S, MW 467.1346, Rdt 70%, Rf = 0.53 (Hex/AcOEt, 0.6:0.4): Melting Point = 273 °C. **Infrared** 3115 (C–H), 1615 (C = N), 1571 (C = C Ar), 1182 (C–N), 1064 (C–S–C), 818 (Ar), 739 (Ar–Br). **^1^HNMR:** 8.33 (d 2H orto, J = 7.32 Hz); 8.10 (d 2H meta, J = 7.32 Hz); 8.11 (s 1H CH); 7.74 (d 1H Ar J = 8.1); 7.63 (d 1H Ar J = 8,2); 7.67 (d 1H, J = 8.2). ^13^ **CNMR:** 148; 126; 127; 140; 175; 161; 129; 133; 129; 137; 162. **HRMS^+^**, calculated: 464.8782, found: 466.8657.

### *N*-(3-methoxybenzylidene)-4–(4-bromophenyl)thiazol-2-imine (Ju-514)

Chemical Formula: C_17_H_13_BrN_2_OS, Molecular Weight: 373.2669. Rdt 70%, Rf = 0.53 (Hex/AcOEt, 0.6:0.4): MP 273–274 °C. **Infrared**: 3114 (C–H), 1615 (C = N), 1576 (C = C Ar), 1180 (C–N), 1063 (C–S–C), 815 (Ar), 740 (Ar–Br). **^1^HNMR:** 8.34 (d 2H orto, J = 7.30 Hz); 8.10 (d 2H meta, J = 7.32 Hz); 8.10 (s 1H CH); 7.74 (d 1H Ar J = 8.1); 7.64 (d 1H Ar J = 8,1); 7.64(d 1H, J = 8.2). ^13^ **CNMR:** 147; 124; 126; 141; 175; 160; 1 133; 129; 138;162. **HRMS^+^**, calculated: 371.9933, found: 371.9630.

### *N*-(2-nitrobenzylidene)-4–(4-bromophenyl)thiazol-2-imine (Ju-516)

Chemical Formula: C_16_H_10_BrN_3_O_2_S, Molecular Weight: 388.2385, yield 53%, Rf = 0.531 (Hex/AcOEt, 0.6:0.4). MP 233–234 °C. **Infrared** 3115 (C–H), 1615 (C = N), 1575 (C = C Ar), 1181 (C–N), 1066 (C–S–C), 817 (Ar), 740 (Ar–Br). **^1^HNMR:** 8.34 (d 2H orto, J = 7.32 Hz); 8.11 (d 2H meta, J = 7.32 Hz); 8.11 (s 1H CH); 7.74 (d 1H Ar J = 8.1); 7.63 (d 1H Ar J = 8,1); 7.66 (d 1H, J = 8.2). ^13^ **CNMR:** 147; 124; 127; 141; 177; 160; 130; 133; 129; 138;163. **HRMS^+^**, calculated: 385.9725., found: 386.9678.

### *N*-(3-nitrobenzylidene)-4–(4-bromophenyl)thiazol-2-imine (Ju-517)

Chemical Formula: C_16_H_10_BrN_3_O_2_S, Molecular Weight: 388.2385, yield 55%, Rf = 0.55 (Hex/AcOEt, 0.6:0.4). MP 155–156 °C. **Infrared** 3116 (C–H), 1614 (C = N), 1575 (C = C Ar), 1180 (C–N), 1067 (C–S–C), 817 (Ar), 740 (Ar–Br). **^1^HNMR:** 8.33 (d 2H orto, J = 7.321 Hz); 8.10 (d, 2H meta, J = 7.32 Hz); 8.12 (s 1H CH); 7.76 (d 1H Ar J = 8.1); 7.63 (d 1H Ar J = 8,1); 7.66 (d 1H, J = 8.2). ^13^ **CNMR:** 148; 124; 126; 140; 178; 161; 130; 132; 129; 137; 163. **HRMS^+^**, calculated: 387.9725, found: 385.9688.

### *N*-(2-fluorobenzylidene)-4–(3-nitrophenyl)thiazol-2-imine (Ju-546)

Chemical Formula: C_16_H_10_FN_3_O_2_S, Molecular Weight: 327.3329, yield 80%, Rf = 0.6 (Hex/AcOEt, 0.6:0.4). MP 182–183 °C. **Infrared:** 3116 (C–H), 1614 (C = N), 1575 (C = C Ar), 1230 (Ar–F), 1180 (C–N), 1068 (C–S–C), 818 (Ar). **^1^HNMR:** 8.29 (d 2H orto, J = 7.28 Hz); 8.12 (d 2H meta, J = 7.30 Hz); 8.11 (s 1H CH); 7.76 (d 1H Ar, J = 8.3); 7.64 (d 1H Ar, J = 8,1); 7.67 (d 1H, J = 8.12). ^13^ **CNMR:** 149; 121; 124; 138; 179; 163; 132; 131; 130; 138; 164. **HRMS^+^**, calculated: 326.0525, found: 325.9786.

### *N*-(3-fluorobenzylidene)-4–(3-nitrophenyl)thiazol-2-imine (Ju-547)

Chemical Formula: C_16_H_10_FN_3_O_2_S, Molecular Weight: 327.3329, yield 63%, Rf = 0.53 (Hex/AcOEt, 0.35:0.65). MP 118.4–119.5 °C. **Infrared:** 3117 (C–H), 1612 (C = N), 1571 (C = C Ar), 1241 (Ar–F), 1183 (C–N), 1069 (C–S–C), 820 (Ar). **^1^HNMR:** 8.31 (d 2H orto, J = 7.28 Hz); 8.11 (d 2H meta, J = 7.29 Hz); 8.09 (s 1H CH); 7.73 (d 1H Ar J = 8.11); 7.64 (d 1H Ar J = 8,0); 7.67 (d 1H, J = 8.12). ^13^ **CNMR:** 149; 121; 124; 137; 179; 168; 133; 131; 129; 138; 162. **HRMS^+^**, calculated: 326.3448, found: 325.9581.

### *N*-(2-hydroxybenzylidene)-4–(3-nitrophenyl)thiazol-2-imine (Ju-551)

Chemical Formula: C_16_H_11_N_3_O_3_S, Molecular Weight: 325.3418, yield 63%, Rf = 0.45 (Hex/AcOEt, 0.5:0.5). MP 118.4–119.5 °C. **Infrared:** 3340 (O–H), 3118 (C–H), 1612 (C = N), 1572 (C = C Ar), 1182 (C–N), 1070 (C–S–C), 820 (Ar). **^1^HNMR:** 8.30 (d 2H orto, J = 7.27 Hz); 8.09 (d 2H meta, J = 7.29 Hz); 8.08 (s 1H CH); 7.72 (d 1H Ar J = 8.11); 7.65 (d 1H Ar J = 8,1); 7.68 (d 1H, J = 8.12). ^13^ **CNMR:** 149; 120; 123; 137; 179; 166; 133; 132; 130; 138; 161. **HRMS^+^**, calculated: 325.0519, found: 326.0537.

### *N*-(3-hydroxybenzylidene)-4–(3-nitrophenyl)thiazol-2-imine (Ju-552)

Chemical Formula: C_16_H_11_N_3_O_3_S, Molecular Weight: 325.3418, yield 59%, Rf = 0.46 (Hex/AcOEt 0.5:0.5). MP 187.5 °C. **Infrared:** 3338 (O–H), 3120 (C–H) 1613 (C = N), 1574 (C = C Ar), 1181 (C–N), 1072 (C–S–C), 821 (Ar). **^1^HNMR:** 8.31 (d 2H orto, J = 7.26 Hz); 8.08 (d 2H meta, J = 7.29 Hz); 8.08 (s 1H CH); 7.72 (d 1H Ar J = 8.11); 7.65 (d 1H Ar J = 8,11); 7.68 (d 1H, J = 8.12). ^13^ **CNMR:** 149; 121; 123; 137; 180; 166; 131; 132; 130; 136; 160. **HRMS^+^**, calculated: 324.0569, found: 325.9581.

### *N*-(2-methylbenzylidene)-4–(3-nitrophenyl)thiazol-2-imine (Ju-555)

Chemical Formula: C_17_H_13_N3O_2_S, Molecular Weight: 323.369, yield 61%, Rf = 0.56 (CH_2_Cl_2_/MeOH 0.98:0.02). MP 213–214 °C. **Infrared:** 3118 (C–H), 1613 (C = N), 1572 (C = C Ar), 1181 (C–N), 1070 (C–S–C), 821 (Ar). **^1^HNMR:** 8.29 (d 2H orto, J = 7.27 Hz); 8.11 (d 2H meta, J = 7.29 Hz); 8.08 (s 1H CH); 7.71 (d 1H Ar J = 8.11); 7.64 (d 1H Ar J = 8,1); 7.67 (d 1H, J = 8.12). ^13^ **CNMR:** 149; 121; 123; 137; 179; 167; 133; 132; 131; 138; 160. **HRMS^+^**, calculated: 322.3810, found: 324.9580.

### Biology

#### *Lb*SOD *heterologous expression and chromatographic purification*

*E. coli* BL21 (DE3) cell carrying the pETM11-*Lb*SOD plasmid were cultured at 37 °C/180 rpm in Luria Bertani medium supplemented with 30 μg/mL kanamycin until OD600 reached 0.6–0.9. At this moment, 1 mM (final concentration) isopropyl-β-D-thiogalactoside (IPTG) was added to the culture and the temperature was reduced to 20 °C. After 16 h, the cells were harvested by centrifugation (8000 rpm -SN93-10, 4 °C, 30 min) and resuspended in lysis buffer (PBS 50 mM, NaCl 100 mM pH 7) supplemented with 1 mM phenylmethanesulphonylfluoride (PMSF). Next, cells were incubated with Lysozyme 0.5 mg/mL, for 30 min and then disrupted by sonication (10 × 15 s bursts with 30 s intervals between each burst, 9 Watts). These steps were carried out in ice-bath. The soluble fraction was clarified by centrifugation (15000 rpm, 20 min, 4 °C) and then loaded onto a HisTrap HP column (GE Healthcare), pre-equilibrated with PBS 50 mM, NaCl 100 mM, 20 mM Imidazole pH 7.0.

The column was washed with 20 column volumes of PBS 50 mM, 100 mM NaCl, 20 mM Imidazole pH 7.0 and then steps of increasing concentration of imidazole (50–500 mM) were used. Purification was monitored by UV absorbance measurement at 280 nm and the level of protein purity was confirmed by sodium dodecyl sulphate-polyacrylamide gel electrophoresis (SDS-PAGE) 12%. The protein fraction was dialyzed in PBS 50 mM, NaCl 100 mM pH 7 with *Amicon Ultra* centrifugal filters (10KDa MWCO, Millipore), 4000 rpm at 4 °C.

Protein concentrations were determined spectrophotometrically using a theoretical extinction coefficient of 55775 M ^− 1 ^cm^−1^ at 280 nm calculated using ExPASy ((http://web.expasy.org/protparam/).

The enzyme was subjected to TEV (*Tobacco Etch Virus*) protease digestion (1 mg per 20 mg *Lb*SOD), 4 °C overnight. After the proteolysis step, *Lb*SOD was loaded again onto His-Trap column to separate cleaved and uncleaved His-tag *Lb*SOD from TEV protease. The column was pre-equilibrated with PBS 50 mM, NaCl 100 mM pH 7 and then the elution of cleaved *Lb*SOD was performed with 10 column volumes of this same buffer. The purification fractions were analyzed by UV measurement at 280 nm and gel electrophoresis (SDS-PAGE 12%) to confirm purification of the protein. The cleaved His-tag *Lb*SOD was concentrated (10KDa MWCO Amicon Ultra devices, Millipore, Burlington, MA, USA) to 10 mg/mL and stored in 30% glycerol at −80 °C.

### Thermal shift assays (TSA)

The assays were carried out in the RT-PCR Applied Biosystems 7500 (Applied Biosystems, Foster City, CA, USA), in triplicate, using 96-well PCR plate (PCR plates 96 well BioRad^®^, Hercules, CA, USA), sealed with transparent capping strips (Flatcap strips BioRad^®^, Hercules, CA, USA). The plates were centrifuged for 2 min, 2000 rpm, 25 °C. After the plates were heated from 25 to 85 °C in increments of 1 °C per minute and fluorescence signal was monitored with SYPRO Orange^®^ dye, with 492 (excitation) and 610 nm (emission) wavelengths.

The results obtained by Applied Biosystems 7500 proprietary software (v2.0) were submitted to processing and analysis in Excel 2007 worksheet (ftp://ftp.sgc.ox.ac.uk/pub/biophysics).

The melting temperature (Tm) values were calculated by non-linear fitting of the melting curves to a Boltzmann sigmoidal function, using GraphPad Prism version 5.0 for Windows (GraphPad^®^ Software, San Diego, CA, USA, www.graphpad.com). Comparisons between Tm of different conditions (ΔTm) were considered as significantly different when *p* < .05, according to the Kruskal–Wallis test followed by Dunn's post-test for multiple comparisons.

#### TSA optimization

Conditions were optimized to *Lb*SOD: protein concentration, buffer, and DMSO concentration. The protein concentration was evaluated (1–5 µM) in the presence of SYPRO Orange^®^ (Thermofisher, Waltham, MA, USA) (1:100 dilution) in ultrapure water qsp 20 µL. Then, the buffers (sodium citrate, sodium phosphate, tris-HCl, and glycine) and pHs (4–9) were evaluated at a final concentration of 50 mM for each buffer, SYPRO Orange^®^ (1: 100 dilution) and *Lb*SOD. Finally, the influence of DMSO (2.5, 5, 10% *v/v*) on the thermal stability of the protein was investigated.

#### Screening of thiazoles derivatives by TSA

The effect of the thiazoles derivatives over the melting temperature of *LbSOD* was evaluated at a single concentration (50 μM final concentration) and an equivalent volume of DMSO was employed as a control (ΔTm = 0.0). Briefly, *Lb*SOD (5 μM) was diluted in 50 mM sodium phosphate buffer (pH 7.0) supplemented with 100 mM NaCl and SYPRO Orange^®^ (1:100 dilution). The 96 well plates were heated from 25 to 85 °C in increments of 1 °C per minute. The results were analyzed as described in the previous section.

### Fluorescent protein-labeled assays

*Lb*SOD (2 mg/mL) was incubated with fluorescein-5-isothiocyanate (FITC*)* dye (10 mg/mL) for 2 h, at 25 °C, with continuous stirring. Then, the solution was loaded on a Hi-Trap HP desalting column (GE Healthcare), previously equilibrated with 50 mM sodium phosphate buffer (pH 8) and 1.5 column volumes of the buffer were injected. The absorbance of the collected fractions was monitored at 280 nm and 490 nm, so correct to the contribution of dye to Absorbance280nm could be calculated according to Equation (1):

(1) Aprotein= A280 – Amax (CF), CF= 0.3 (Abs fluorescein at 280 nm)

Aprotein stands for the protein absorbance after correction; A280 is the absorbance at 280 nm of the purified sample; Amax: maximal dye absorption at 280 nm or correction factor (CF) corresponding to 0.3 to fluorescein.

Next, *Lb*SOD was diluted to 5 µM and the influence of putative inhibitors (50 µM) over its fluorescence was measured at 25 °C for 10 min on an Applied Biosystems 7500 RT-PCR machine. The experiments were carried out in triplicate using 96-well PCR plates (PCR plates 96 well BioRad^®^). Fluorescence data was recorded on the Applied Biosystems 7500 Software v2.0 and then analyzed on GraphPad Prism V5.0 software (La Jolla, CA, USA).

### Isothermal titration calorimetry

ITC experiments were carried out in a Microcal VP-ITC Model, at 298 K. All the solutions were thoroughly degassed prior to the titrations to avoid the formation of bubbles during the experiment. The reference cell contained distilled water. The sample cell was filled with *Lb*SOD (20 µM) diluted in sodium phosphate buffer 0.05 M pH 7 with DMSO (5%*v/v*). The injection syringe was loaded with ligand 400 µM (previously dissolved in the same solution as the protein). Control experiments were carried out with sodium phosphate buffer 0.05 M pH 7 and DMSO (5%*v/v*) in the sample cell to determine the dilution heat. Injections were started after baseline stability had been achieved. The ligands were titrated in the sample cell through 21 injections of 13 µL and the first injection of 10 µL. The solutions of the sample cell were stirred during the experiment at 200 rpm to ensure mixing.

The heat variation, following each injection of the ligand in the sample cell, were employed to build the titration isotherms, as available in Origin package supplied with the calorimeter.

The thermodynamic parameters ΔG (Gibbs free energy), ΔS (entropy), and ΔH (enthalpy) were calculated using the Equation (2):

(2) ΔG = ΔH – TΔS

Where R is the universal gas constant, T is the temperature (Kelvin).

## Results and discussion

Previous studies have shown that 1,3-thiazole derivatives have leishmanicidal activity^26^ and that phthalimide-thiazole derivatives have higher affinity to *L. infantum* FeSOD than human CuZnSOD^27^. These results suggest the thiazole ring is a suitable scaffold upon which novel SOD inhibitors might be developed. Hence, we designed a series of 2,4 substituted thiazole derivatives as potential *Lb*SOD inhibitors

### Synthesis of thiazole derivatives

**Scheme 1.**
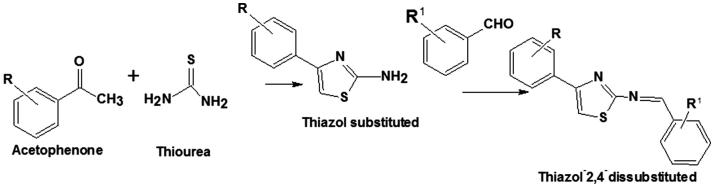
The synthesis of 2-aminothiazole and Schiff base derivatives.

The starting material 2-aminothiazole-4-substituted Ju-436 and Ju-533 were prepared, as described previously,^28^ with minor modifications. The condensation of substituted benzaldehydes in position 2 was carried out to obtain the respective Schiff bases, as outlined in Scheme 1. The spectroscopic data showed the formation of both starting material and Schiff bases, with characteristic ^1^HNMR and ^13^CNMR absorption of CH = N between 4.92–4.04 and 171.76–168.25 ppm, respectively. The stereochemistry of CH = N bond is assumed to be *E*, as reported in literature for thiazole Schiff bases derivatives^29^.

### *Expression and purification Lb*SOD

*E. coli* Bl21 (DE3) cells were transformed with plasmid *Lb*SOD-pET-M11 and *Lb*SOD expression was performed with IPTG 1 mM at 20 °C for 16 h.

**Figure 1.**
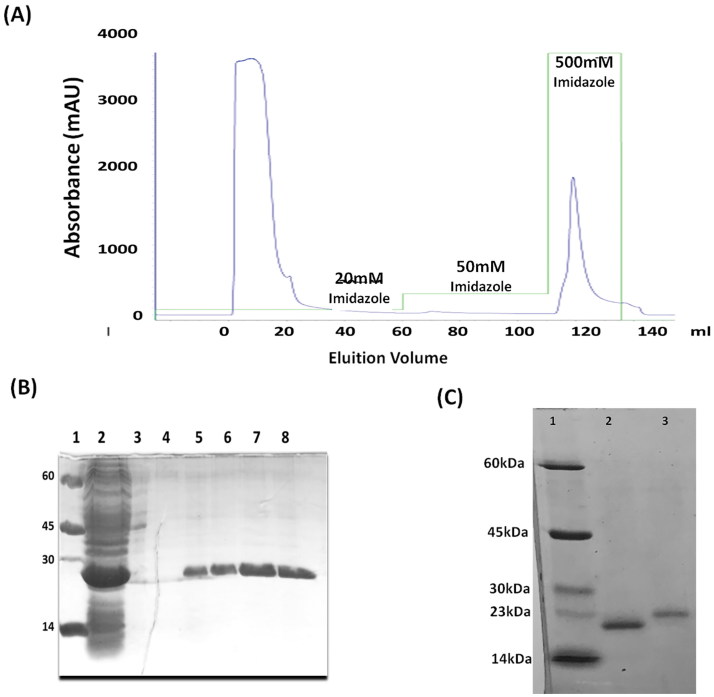
Results of *Lb*SOD purification: (A) purification chromatogram using 20–500 mM imidazole gradient. (B) SDS-PAGE with samples of the *Lb*SOD (23 kDa). 1: Molecular weight standard (kDa); 2: supernatant purification; 3: Fraction 20 mM Imidazole; 4: 50 mM Imidazole; 5–8: 500 mM imidazole. (C) SDS-PAGE with samples of clivage *Lb*SOD (1) and 6xHis-tag *Lb*SOD(2).

The LIC vector employed in this work, pETM11, was constructed with *6xhistidine* tag, which allows the expression of the His-tag fused *Lb*SOD and its purification by affinity chromatography. The purification was based on the interaction of histidine residues with nickel ions immobilized on the chromatographic column (Ni-sepharose) and *Lb*SOD was eluted in a higher concentration of imidazole (500 mM) (Figure 1(A); (B)).

Once the His-tag can alter kinetics and structural features of *Lb*SOD, the biological assays were carried out after the tag was removed by proteolytic cleavage with TEV protease (Tobacco etch virus) (Figure 1(C)). The overall yield of the purification steps is 20 mg of *Lb*SOD per liter of culture medium.

Once the pure *Lb*SOD was obtained, the next step consisted the identification of potential inhibitors (Table 1). Considering that thiazole derivatives interfere with the kinetic assay of *Lb*SOD due to the absorption of light at the same wavelength of the assay (320 nm) (data not shown), the thermal shift assays (TSA) and fluorescent protein-labeled assays (FPLA) were employed to evaluate the potential *Lb*SOD inhibitors.

**Table 1.** Thiazole derivatives evaluated against *Lb*SOD.


Compound	R	R_1_
Ju-436	4-bromo	H
Ju-445	4-bromo	3-methoxy
Ju-450	4-nitro	2-bromo
Ju-480	4-nitro	3,4-dibromo
Ju-514	4-bromo	3-methoxy
Ju-516	4-bromo	2-nitro
Ju-517	4-bromo	3-nitro
Ju-533	3-nitro	H
Ju-546	3-nitro	2-fluor
Ju-547	3-nitro	3-fluor
Ju-551	3-nitro	2-hidroxy
Ju-552	3-nitro	3-hidroxy
Ju-555	3-nitro	2-methyl
Ju-567	3-nitro	H

### Thermal shift assays (TSA)

In general, TSA is used to identify the conditions and molecules that influence protein stability^23^. This assay relies on the interaction of a fluorophore with hydrophobic regions of the protein, which are exposed as a result of thermal denaturation^30^. The change in fluorescence is a function of the temperature increase, and, consequently, protein unfolding. Thus, it is possible to calculate the mean thermal transition point (Tm: melting temperature)^31^ and thus to observe the effect of different buffers, additives, and/or ligands on a protein^32^ ^,^ ^33^. The increase in protein stability is related to an increase in the conformational homogeneity of the protein sample. Therefore, the identification of ideal conditions, such as the buffer and pH used in the assay, is of great relevance to assay standardization^34^ ^,^ ^35^ Accordingly, optimal conditions of protein stability were probed by thermal shift assays, varying conditions of concentration de *Lb*SOD, buffer, pH, and organic solvent (DMSO), as described next.

### Biological assay standardization

**Figure 2.**
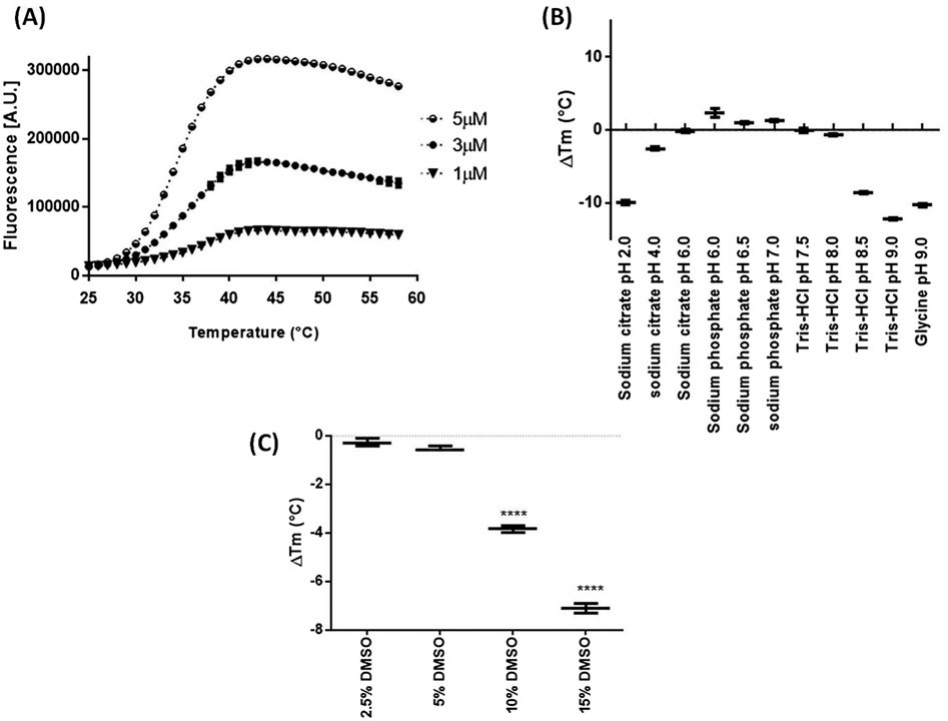
Optimization of *Lb*SOD thermal shift assay parameters. (A) Effect *of LbSOD* concentration over the malting curve; effect of pH, (B) and DMSO, (C) over *Lb*SOD thermal stability; **** = *p* < .001.

In TSA, a linear increase of the detected signal is observed as a function of the protein concentration: increase the number of sites available for interaction with the dye and consequently, the displayed signal^36^. However, high concentrations of protein may lead to aggregation and errors in the interpretation of the results^30^. The TSA curves at different *Lb*SOD concentrations (Figure 2(A)), depict the expected signal-to-noise improvement as the concentration increases and at 5 µM, it exhibits a well-defined onset and final transition that was considered suitable for the next standardization steps.

Comparison of *Lb*SOD stability at different buffers and pHs suggests that it is stable between pHs 4.0 and 8.0, as ΔTm values do not exhibit significant statistical differences (*p* > .05) in this range (Figure 2(B)). Either below (i.e. pH 2.0) or above this range (i.e. pH 8.5 – 9) *Lb*SOD stability decreases. MEIER et al. (1995) showed that *Propionibacterium shermanha* Fe-SOD activity decreases at pH <5 (cytochrome C indirect assay; xanthine/xanthine oxidase system)^37^. Similarly, the activity of FeSOD from *Plasmodium vickey* also decreases at pH <6.0^16^ ^,^ ^38^. Considering the reported data and the results described above, pH 7.0 was chosen for subsequent assays.

The organic solvent used to solubilize the inhibitors may affect the stability of the enzyme by influencing the formation of intermolecular interactions, such as hydrogen bonds and hydrophobic interactions that stabilize the three-dimensional structure of the enzyme^39^. Then, the effect of DMSO on the thermal stability of *Lb*SOD was also investigated. The assessment of different DMSO concentrations suggests that up to 5% (*v/v*) of DMSO does not significantly affect the thermal stability of *Lb*SOD (*p* > .05) (Figure 2(C)) and, for that reason, this is the concentration of DMSO used in the assays.

### Screening of thiazole derivatives

**Figure 3.**
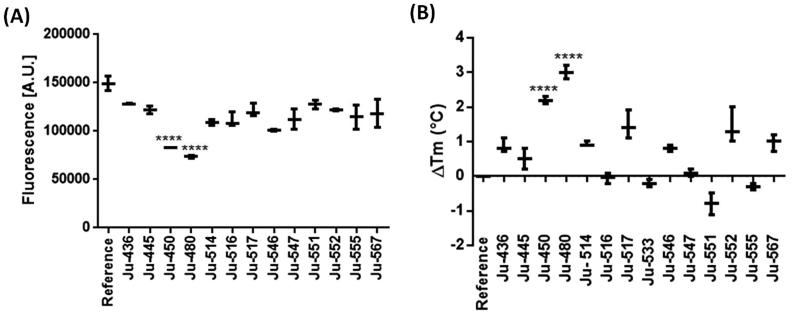
Screening of thiazol derivatives as *Lb*SOD putative binders at 50 µM. (A) FITC-labeled fluorescence assay. (B) Thermal shift assay. **** = *p* < .001 (compared to control).

HTS methods afford false-positive compounds due to a number of reasons^40^, but it is a consensus that data from single-concentration experiments is a ubiquitous shortcoming of this strategy^41^. The fact that several SOD assays rely on indirect methods that are subject to interference by oxi-redoxi conditions makes this concern an ordinary problem for *Lb*SOD hit identification campaigns. In order to increase the number of true-positives, it is common practice to employ a counter-screening method, whose end-point is different from the first screening assay. Accordingly, the first screening method described in this work relies on fluorescein-5-isothiocyanate (FITC) covalent binding to the target protein^42^. Once *Lb*SOD was labeled with FITC, any change in the protein conformation would lead to a change in the fluorescence. In case thiazole derivatives cause a conformational shift that exposes FITC-labeled residues, an increased fluorescence signal would be observed. On the other hand, if ligand binding hides the FITC-labeled residues, a decreased signal is expected. Then, the conformational modification due to thiazole derivatives binding to *Lb*SOD was evaluated at a single concentration. This approach suggests that Ju-450 and Ju-480 are the most promising hits (Figure 3).

When protein labeling occurs far from the *Lb*SOD active site or there is minor-induced fit upon ligand binding (e.g. low entropic cost), the change in the fluorescence signal might be low and promising compounds would be discarded. An even more troublesome scenario would be a covalent modification of a residue that is crucial for binding. Considering all these shortcoming of protein covalent labeling, we resorted to thermal shift assays as a secondary counter-screening approach. Although this strategy also depends on a fluorescent label (Sypro-orange^®^), covalent binding is not an issue anymore.

TSA shows that Ju-514, Ju-517, and Ju-546 stabilize *Lb*SOD (ΔTm ≅ +1.0 °C). However, only Ju-450, Ju-480 have a significant effect over *Lb*SOD thermal stability (*p* < .05). Taking both results into consideration, Ju-450 and Ju-480 would be considered a true hit.

**Figure 4.**
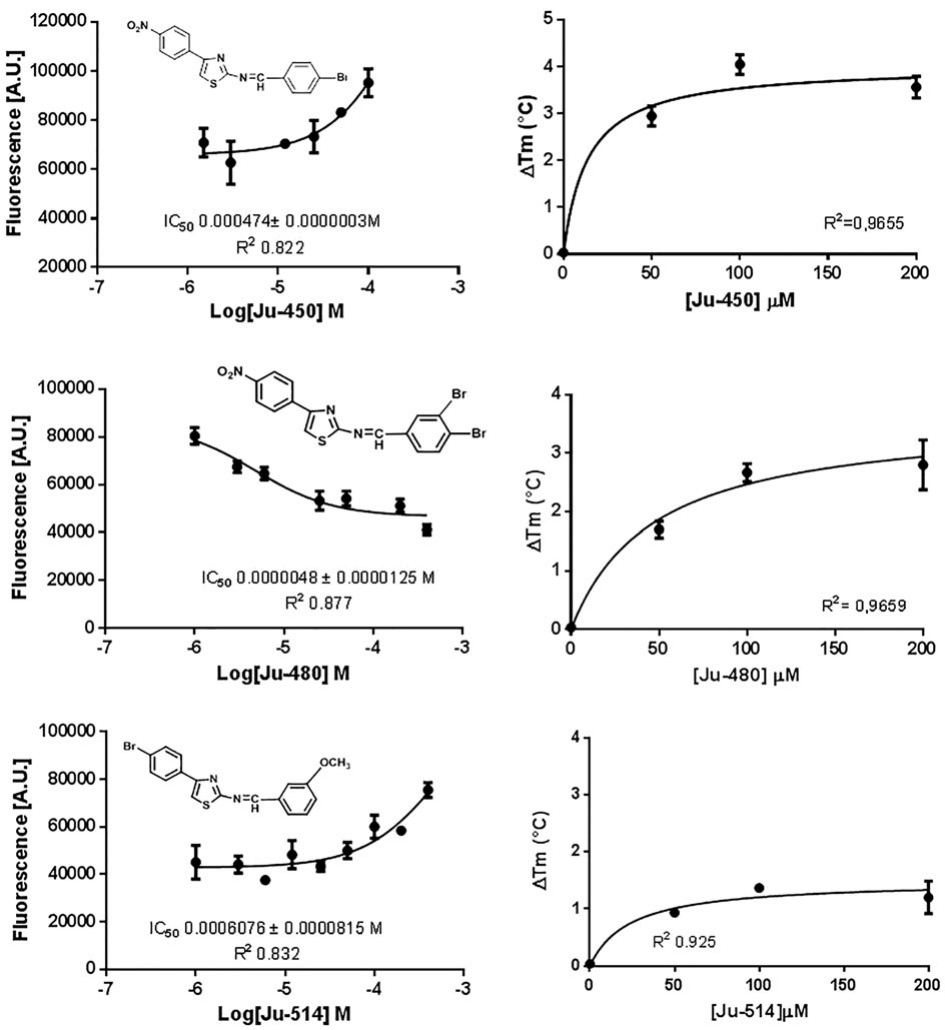
Concentration-response curves for thiazole derivatives. Left-hand side panel – conformational change in FITC-*Lb*SOD due to ligand binding. Right-hand side panel – Thermal stabilization of *Lb*SOD dur to ligand binding.

Both Ju-450 and Ju-480 have a *p*-nitrophenyl moiety at the 4-position of the thiazole ring and increase the fluorescence signal in FLPA. On the other hand, Ju-514 has a *p*-bromophenyl moiety at the equivalent position and reduces the fluorescence signal in the FLPA. Given the overall similarity between these compounds, we decided to include Ju-514 in subsequent assays to make sure no true ligand was excluded from our study (Figure 4).

Although all evaluated compounds exhibited dose-response profile that are typical of true ligands, Ju-450 and Ju-514 have low affinity to *Lb*SOD (Kd= 4.7 ± 0.01 mM and Kd= 6.1 ± 0.8 mM, respectively). Hence, structure-activity relationships based on those compounds would be of little help to design potent *Lb*SOD inhibitors. Instead, we decided to focus our efforts on Ju-480, that shows low-micromolar affinity to the macromolecular target (Kd= 4.8 ± 12.5 μM) and employ a label-free method to study its thermodynamic signature^43^.

### Isothermal titration calorimetry

**Figure 5.**
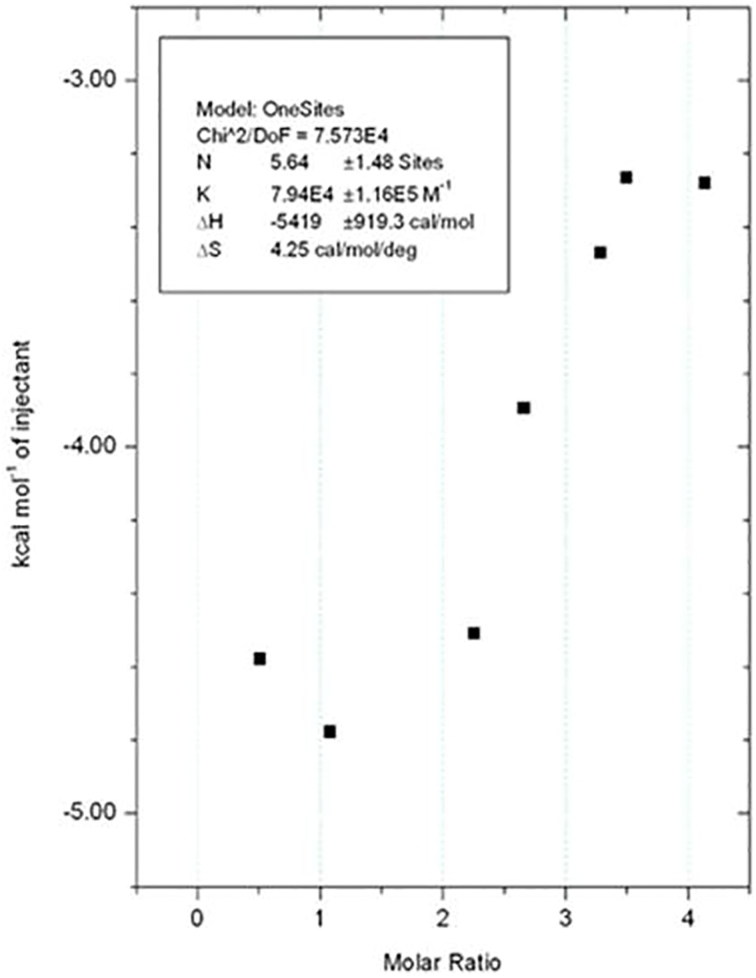
Isothermal titration calorimetry profile of *Lb*SOD with Ju-480, at 298K, in PBS pH 7.

Once ITC measures the heat released or absorbed upon the ligand-macromolecule interaction, it is possible to directly determine thermodynamic parameters ΔH and ΔS^44^. Ju-480 has favorable enthalpic (**Δ**H= −4.22 ± 1.6970 kcal/mol) and entropic (**Δ**S = 0.0037 ± 0.0006 kcal/mol) contributions to affinity (Figure 5). The enthalpic contributions are related to the intermolecular interactions, such as hydrogen bonds, Van der Waals and dipole-dipole interactions, etc. whereas the entropic contributions are, in general, due to binding-site desolvation and/or induced fit upon ligand binding^5^. Therefore, our results suggest that Ju-480 affinity might be improved either by the addition of substituents that interact with hydrophobic patches in *Lb*SOD surface, as was observed in the optimization of HIV-1 protease inhibitors,^45^ or by structure rigidification,^46^ which might reduce the entropic penalty for Ju-480 to adopt the bioactive conformation, as entropic contributions are easier to optimize than enthalpic ones.^43^.

## Conclusion

Although TSA is commonly employed for screening putative ligands of promising targets, the results presented in this work highlight that its use alone might lead to the selection of false-positive *Lb*SOD ligands. On the other hand, the combined use of TSA and FPLA assays lead to the identification of Ju-480, a thiazole derivative that has a low-micromolar affinity to *L. braziliensis* Superoxide dismutase. Label-free methods confirm that Ju-480 binds to LbSOD and suggests that structure rigidification might increase the ligand’s affinity to its macromolecular target.

## References

[1] Patil SR, Asrondkar A, Patil V Antileishmanial potential of fused 5-(pyrazin-2-yl)-4H-1,2,4-triazole-3-thiols: Synthesis, biological evaluations and computational studies. Bioorganic Med Chem Lett 2017;27:3845–50. 10.1016/j.bmcl.2017.06.053 28693910

[2] Torres-Guerrero E, Quintanilla-Cedillo MR, Ruiz-Esmenjaud J, Arenas R Leishmaniasis: a review. F1000Research 2017;6:750 28649370 10.12688/f1000research.11120.1 PMC5464238

[3] Cecílio P, Pérez-Cabezas B, Santarém N, et al Deception and manipulation: the arms of Leishmania, a successful parasite. Front Immunol 2014;5:1–16. 25368612 10.3389/fimmu.2014.00480 PMC4202772

[4] Marchand P, Bazin MA, Pagniez F, et al. Synthesis, antileishmanial activity and cytotoxicity of 2,3-diaryl- and 2,3,8-trisubstituted imidazo[1,2-a]pyrazines. Eur J Med Chem 2015;103:381–95. 26383125 10.1016/j.ejmech.2015.09.002

[5] Romero AH, Medina R, Alcala A, et al. Design, synthesis, structure-activity relationship and mechanism of action studies of a series of 4-chloro-1-phthalazinyl hydrazones as a potent agent against *Leishmania braziliensis* . Eur J Med Chem 2017;127:606–20. 28119201 10.1016/j.ejmech.2017.01.022

[6] Haldar AK, Sen P, Roy S Use of antimony in the treatment of Leishmaniasis: current status and future directions. Mol Biol Int 2011;2011:1–23. 10.4061/2011/571242 PMC3196053 22091408

[7] Bezerra de Menezes JP, Guedes CES, Petersen AL, et al. Advances in development of new treatment for Leishmaniasis. Biomed Res Int 2015;2015:1–12. 10.1155/2015/815023 PMC4442256 26078965

[8] Castro M. d M, Cossio A, Velasco C, et al. Risk factors for therapeutic failure to meglumine antimoniate and miltefosine in adults and children with cutaneous leishmaniasis in Colombia: a cohort study. PLoS Negl Trop Dis 2017;1:e0005515. 10.1371/journal.pntd.0005515 PMC5393627 28379954

[9] Vanaerschot M, de Doncker S, Rijal S, et al. Antimonial resistance in *Leishmania donovani* is associated with increased *in vivo* parasite burden. PLoS One 2011;6:1–5. 10.1371/journal.pone.0023120 PMC3148249 21829701

[10] Rubiano LC, Miranda MC, Muvdi Arenas S, et al. Noninferiority of miltefosine versus meglumine antimoniate for cutaneous leishmaniasis in children. J Infect Dis 2012;205:684–92. 22238470 10.1093/infdis/jir816 PMC3266136

[11] Turrens JF Oxidative stress and antioxidant defenses: a target for the treatment of diseases caused by parasitic protozoa. Mol Aspects Med 2004;25:211–20. 15051329 10.1016/j.mam.2004.02.021

[12] Handy DE, Loscalzo J Redox regulation of mitochondrial function. Antioxid Redox Signal 2012;16:1323–67. 22146081 10.1089/ars.2011.4123 PMC3324814

[13] Tomás AM, Castro H Redox Metabolism in Mitochondria of Trypanosomatids. Antioxid Redox Signal 2013;19:696–707. 23025438 10.1089/ars.2012.4948 PMC3739956

[14] Fukai T, Ushio-Fukai M Superoxide dismutases: role in redox signaling, vascular function, and diseases. Antioxid Redox Signal 2011;15:1583–606. 21473702 10.1089/ars.2011.3999 PMC3151424

[15] Sanz AM, Gómez-Contreras F, Navarro P, et al. Efficient inhibition of iron superoxide dismutase and of Trypanosoma cruzi growth by benzo[g]phthalazine derivatives functionalized with one or two imidazole rings. J Med Chem 2008;51:1962–6. 18293910 10.1021/jm701179m

[16] Prakash K, Goyal M, Soni A, et al. Molecular cloning and biochemical characterization of iron superoxide dismutase from the rodent malaria parasite Plasmodium vinckei. Parasitol Int 2014;63:817–25. 25091832 10.1016/j.parint.2014.07.004

[17] Miller A-F Superoxide dismutases: active sites that save, but a protein that kills. Curr Opin Chem Biol 2004;8:162–8. 15062777 10.1016/j.cbpa.2004.02.011

[18] O'Shea IP, Shahed M, Aguilera-Venegas B, Wilkinson SR Evaluating 5-nitrothiazoles as trypanocidal agents. Antimicrob Agents Chemother 2016;60:1137–40. 26596950 10.1128/AAC.02006-15 PMC4750693

[19] Papadopoulou MV, Bloomer WD, Rosenzweig HS, et al. European Journal of Medicinal Chemistry Antitrypanosomal activity of 5-nitro-2-aminothiazole-based compounds. Eur J Med Chem 2016;117:179–86. 27092415 10.1016/j.ejmech.2016.04.010 PMC4876673

[20] Sanchez-Moreno M, Gomez-Contreras F, Navarro P Imidazole-containing phthalazine derivatives inhibit Fe-SOD performance in Leishmania species and are active in vitro against visceral and mucosal leishmaniasis. Parasitology 2015;142:1350. 26054948 10.1017/S0031182015000657

[21] Romero AH, López SE In silico molecular docking studies of new potential 4-phthalazinyl-hydrazones on selected *Trypanosoma cruzi* and Leishmania enzyme targets. J Mol Graph Model 2017;76:313–29. 28763686 10.1016/j.jmgm.2017.07.013

[22] Boivin S, Kozak S, Meijers R Optimization of protein purification and characterization using Thermofluor screens. Protein Expr Purif 2013;91:192–206. 23948764 10.1016/j.pep.2013.08.002

[23] Senisterra G, Chau I, Vedadi M Thermal denaturation assays in chemical biology. Assay Drug Dev Technol 2012;10:128–36. 22066913 10.1089/adt.2011.0390

[24] Zubrienė A, Kazlauskas E, Baranauskienė L, et al. Isothermal titration calorimetry and thermal shift assay in drug design. Eur Pharm Rev 2011;16 https://www.europeanpharmaceuticalreview.com/article/7622/isothermal-titration-calorimetry-and-thermal-shift-assay-in-drug-design/

[25] Hungerford G, Benesch J, Mano JF, Reis RL Effect of the labelling ratio on the photophysics of fluorescein isothiocyanate (FITC) conjugated to bovine serum albumin. Photochem Photobiol Sci 2007;6:152–8. 17277838 10.1039/b612870j

[26] Rodrigues CA, Freire P, Oliveira M, et al. 4-Phenyl-1,3-thiazole-2-amines as scaffolds for new antileishmanial agents. J Venom Anim Toxins Incl Trop Dis 2018;24:26. 10.1186/s40409-018-0163-x PMC6131760 30214457

[27] Aliança AS. d S, Oliveira AR, Feitosa APS, et al. *In vitro* evaluation of cytotoxicity and leishmanicidal activity of phthalimido-thiazole derivatives. Eur J Pharm Sci 2017;105:1–10. 28478133 10.1016/j.ejps.2017.05.005

[28] Vinícius M, Souza ND, Ferreira SB, et al. Métodos de obtenção e aplicações sintéticas de tiazóis, uma importante classe de compostos heterocíclicos. Química Nova 2005;28:77–84.

[29] Moustafa SA, Ali MM, El-rashedy AA Synthesis, anticancer activity and molecular docking study of Schiff base complexes containing thiazole moiety. BJBAS 2016;5:85–96.

[30] Reinhard L, Mayerhofer H, Geerlof A, et al. Optimization of protein buffer cocktails using Thermofluor. Acta Crystallogr Sect F Struct Biol Cryst Commun 2013;69:209–14. 10.1107/S1744309112051858 PMC3564630 23385769

[31] Matulis D, Kranz JK, Salemme FR, Todd MJ Thermodynamic stability of carbonic anhydrase: Measurements of binding affinity and stoichiometry using thermofluor. Biochemistry 2005;44:5258–66. 15794662 10.1021/bi048135v

[32] Ericsson UB, Hallberg BM, DeTitta GT, et al. Thermofluor-based high-throughput stability optimization of proteins for structural studies. Anal Biochem 2006;357:289–98. 16962548 10.1016/j.ab.2006.07.027

[33] Lo MC, Aulabaugh A, Jin G, et al. Evaluation of fluorescence-based thermal shift assays for hit identification in drug discovery. Anal Biochem 2004;332:153–9. 15301960 10.1016/j.ab.2004.04.031

[34] Leite FHA, Santiago PBG. d S, Froes TQ, et al. Structure-guided discovery of thiazolidine-2,4-dione derivatives as a novel class of Leishmania major pteridine reductase 1 inhibitors. Eur J Med Chem 2016;123:639–48. 27517809 10.1016/j.ejmech.2016.07.060

[35] Lacerda A, Teles B, Silva RR, et al. Identification, characterization and molecular modelling studies of *Schistosoma mansoni* dihydrofolate reductase inhibitors: from assay development to hit identification. Curr Topics Med Chem 2018;18:1–12. 10.2174/1568026618666180509150134 29741139

[36] Venkatraman J, Bhat J, Solapure SM, et al. Screening, identification, and characterization of mechanistically diverse inhibitors of the *Mycobacterium tuberculosis* enzyme, pantothenate kinase (CoaA). J Biomol Screen 2012;17:293–302. 22086722 10.1177/1087057111423069

[37] Meier B, Michel C, Saran M, et al. Kinetic and spectroscopic studies on a superoxide dismutase from *Propionibacterium shermanii* that is active with iron or manganese: pH-dependence. Biochem J 1995;310:945–50. 7575431 10.1042/bj3100945 PMC1135987

[38] Sheng Y, Abreu IA, Cabelli DE, et al. Superoxide dismutases and superoxide reductases. Chem Rev 2014;114:3854–918. 24684599 10.1021/cr4005296 PMC4317059

[39] Arakawa T, Kita Y, Timasheff SN Protein precipitation and denaturation by dimethyl sulfoxide. Biophys Chem 2007;131:62–70. 17904724 10.1016/j.bpc.2007.09.004

[40] Zhang JH, Chung TDY, Oldenburg KR A simple statistical parameter for use in evaluation and validation of high throughput screening assays. J Biomol Screen 1999;4:67–73. 10838414 10.1177/108705719900400206

[41] de Araujo ED, Manaswiyoungkul P, Israelian J, et al. High-throughput thermofluor-based assays for inhibitor screening of STAT SH2 domains. J Pharm Biomed Anal 2017;143:159–67. 28600954 10.1016/j.jpba.2017.04.052

[42] Breen CJ, Raverdeau M, Voorheis HP Development of a quantitative fluorescence-based ligand-binding assay. Sci Rep 2016;6:1–9. 27161290 10.1038/srep25769 PMC4861924

[43] Baron R, McCammon JA Molecular recognition and ligand association. Annu Rev Phys Chem 2013;64:151–75. 23473376 10.1146/annurev-physchem-040412-110047

[44] Linkuvienė V, Krainer G, Chen WY, Matulis D Isothermal titration calorimetry for drug design: precision of the enthalpy and binding constant measurements and comparison of the instruments. Anal Biochem 2016;515:61–4. 27717855 10.1016/j.ab.2016.10.005

[45] Velazquez-campoy A, Todd MJ, Freire E HIV-1 protease inhibitors: enthalpic versus entropic optimization of the binding affinity. Biochemistry 2000;39:2201–7. 10694385 10.1021/bi992399d

[46] Chaires JB Calorimetry and thermodynamics in drug design. Annu Rev Biophys 2008;37:135–51. 18573076 10.1146/annurev.biophys.36.040306.132812

